# ApoE Receptor 2 Regulates Synapse and Dendritic Spine Formation

**DOI:** 10.1371/journal.pone.0017203

**Published:** 2011-02-15

**Authors:** Sonya B. Dumanis, Hyun-Jung Cha, Jung Min Song, Justin H. Trotter, Matthew Spitzer, Ji-Yun Lee, Edwin J. Weeber, R. Scott Turner, Daniel T. S. Pak, G. William Rebeck, Hyang-Sook Hoe

**Affiliations:** 1 Department of Neuroscience, Georgetown University Medical Center, Washington, D.C., United States of America; 2 Department of Pharmacology, Georgetown University Medical Center, Washington, D.C., United States of America; 3 Department of Neurology, Georgetown University Medical Center, Washington, D.C., United States of America; 4 Department of Molecular Pharmacology and Physiology, University of South Florida, Tampa, Florida, United States of America; University of Cincinnatti, United States of America

## Abstract

**Background:**

Apolipoprotein E receptor 2 (ApoEr2) is a postsynaptic protein involved in long-term potentiation (LTP), learning, and memory through unknown mechanisms. We examined the biological effects of ApoEr2 on synapse and dendritic spine formation—processes critical for learning and memory.

**Methodology/Principal Findings:**

In a heterologous co-culture synapse assay, overexpression of ApoEr2 in COS7 cells significantly increased colocalization with synaptophysin in primary hippocampal neurons, suggesting that ApoEr2 promotes interaction with presynaptic structures. In primary neuronal cultures, overexpression of ApoEr2 increased dendritic spine density. Consistent with our *in vitro* findings, ApoEr2 knockout mice had decreased dendritic spine density in cortical layers II/III at 1 month of age. We also tested whether the interaction between ApoEr2 and its cytoplasmic adaptor proteins, specifically X11α and PSD-95, affected synapse and dendritic spine formation. X11α decreased cell surface levels of ApoEr2 along with synapse and dendritic spine density. In contrast, PSD-95 increased cell surface levels of ApoEr2 as well as synapse and dendritic spine density.

**Conclusions/Significance:**

These results suggest that ApoEr2 plays important roles in structure and function of CNS synapses and dendritic spines, and that these roles are modulated by cytoplasmic adaptor proteins X11α and PSD-95.

## Introduction

ApoE receptors are a family of transmembrane proteins that mediate endocytosis of ligands and are then recycled back to the cell surface [1]. ApoE receptors include the LDL receptor, LDL receptor related proteins (LRP-1, LRP-1B, LRP-2), ApoE receptor 2 (ApoEr2), and the very low density lipoprotein receptor (VLDLr). Each of these type I transmembrane receptors has a large N-terminal extracellular domain, with multiple ligand-binding repeats, and small C-terminal cytoplasmic adaptor domains with one or several NPXY sequences for receptor-mediated endocytosis. These ApoE receptors are involved in neuronal migration during brain development [2], influx of calcium through NMDA channels [3], neurite outgrowth [4], LTP and memory [5]. However, the mechanisms by which ApoE receptors affect LTP, learning, and memory are unclear.

ApoE receptors interact with cytoplasmic adaptor proteins via specific binding motifs. ApoEr2 interacts with PSD-95 [5], [6], [7], [8], a major postsynaptic density protein important for synapse formation and function [9], through a domain encoded by the alternatively spliced ApoEr2 exon 19 [10]. This region of ApoEr2 regulates memory and behavior in mice [5]. Recently, we and others have shown that proteins in the X11 family also interact with ApoEr2 via exon 19 [11], [12]. X11 family members (X11α, β and γ, also referred to as mint-1, -2, and -3 for munc interaction) are present at both presynaptic and postsynaptic membranes [13]. Presynaptically, X11α plays essential roles in vesicle docking and exocytosis via interactions with munc and CASK:Veli [14], [15]. X11α is also involved in synapse formation and neuroligation [15], [16]. However, it is unclear how interactions between ApoEr2 and its cytoplasmic adaptor proteins are involved in synapse and dendritic spine formation.

We examined the roles of ApoEr2 in synaptic and dendritic spine structure *in vitro* and *in vivo*. We found that viral expression of ApoEr2 in neuronal culture significantly increased synaptic protein levels (synaptophysin and PSD-95) and that, in a heterologous co-culture system, overexpressed ApoEr2 increased presynaptic differentiation. Overexpression of ApoEr2 also increased dendritic spine density, decreased levels of GluA1 and increased levels of GluA2 in primary neurons, suggesting that ApoEr2 may modulate the AMPA receptor pools at the synapse. Moreover, *in vitro* experimentation with ApoEr2 deletion constructs revealed that the both the extracellular and intracellular domains of ApoEr2 are necessary for increasing dendritic spine density. We also found that overexpressing X11α inhibited the effects of ApoEr2 on synapses and dendritic spines. Conversely, overexpressing PSD-95 enhanced the effects of ApoEr2 on synapses and dendritic spines. These data suggest that ApoEr2's effects in the synapse and on dendritic spines are modulated via potentially competitive interactions with specific cytoplasmic adaptor proteins.

## Materials and Methods

### Mice

ApoEr2 null mice were raised from stocks originally created through targeted-deletion of each individual gene [17]. Wild-type littermates were used as controls for all experiments. The animals were provided a standard rodent chow diet (Diet 7001, Harlan Teklad, Madison, WI) and water ad libitum. All procedures were performed in accordance with the protocols approved by the Institutional Committee for Use and Care of Laboratory Animals of the University of South Florida, under animal protocol number R3336.

### Cell lines and culture conditions

COS7 cells (Lombardi Co-Resources Cancer Center, Georgetown University) were maintained in Opti-MEM® (Invitrogen) with 10% fetal bovine serum (FBS, Life Technologies, Inc.) in a 5% CO_2_ incubator. The cells were transiently transfected with 0.5–1 µg of plasmid in FuGENE 6 (Roche) according to the manufacturer's protocol and cultured 24 hr in DMEM containing 10% FBS. For co-transfections, cells were similarly transfected with 0.5–1 µg of each plasmid in FuGENE 6 (Roche) and cultured 24 hr in DMEM with 10% FBS.

### Antibodies

We used antibodies anti-HA (Abcam), anti-X11α (BD Bioscience, Sigma), anti-Flag (Sigma), anti-PSD-95 (Chemicon), anti-GFP (Invitrogen), β-actin (Chemicon), anti-ApoEr2 (Sigma), anti-GluA1 (Chemicon), anti-GluA2 (Chemicon), anti-c-myc (Abcam), and anti-synaptophysin (Sigma).

### Primary neuron culture and transfection

Hippocampal neurons from embryonic day 18–19 Sprague–Dawley rats were cultured at 150 cells/mm^2^ as described [18]. Neurons were transfected at 12, 14 or 16 days *in vitro* (DIV) with GFP-ApoEr2, ApoEr2-HA, ApoEr2-myc, ApoEr2 deletion constructs with C-terminal myc tag, PSD-95-Flag, X11α-Flag, GFP or empty vector by lipofectamine 2000 (Invitrogen) (2 µg DNA per well) according to manufacturer's instructions. Transcription of each insert was driven by the CMV promoter.

### Immunocytochemistry

Hippocampal cultured neurons were fixed either in 4% paraformaldehyde (for morphological analysis) or in methanol at −20°C for 10 min (for immunostaining of endogenous synaptic markers). Antibodies for immunostaining were incubated in GDB buffer (0.1% gelatin, 0.3% Triton X-100, 16 mM sodium phosphate pH 7.4, 450 mM NaCl). Cell surface expression levels of ApoEr2 were performed as described [19]. Live neuronal cultures were briefly incubated (10 min) with antibodies directed against extracellular N-termini of GFP (10 µg/mL in conditioned medium) to specifically label surface receptors, then lightly fixed for 5 min in 4% paraformaldehyde (non-permeabilizing conditions). After fixation, the surface-remaining antibody labeled GFP was measured with alexa fluor 555–linked anti-mouse secondary antibodies for 1 hr. Immunostaining was quantified using Metamorph analysis of immunostaining intensity or punctate number from Z-stacked images obtained with a Zeiss LSM510 confocal microscope. Surface localization of staining was also confirmed visually from these images. The total HA intensity quantification for figure S2 used image J analysis.

### Neuronal cultures and Sindbis virus infection

Primary hippocampal neurons were prepared as described [18]. Human ApoEr2 or GFP were cloned into the pSinRep5 Sindbis virus vector (Invitrogen) and replication-defective pseudovirions produced according to the manufacturer's directions. Neurons were typically between DIV14-16 at time of infection and the duration of infection was limited to 18–24 hr; no cytotoxicity was apparent during infection.

### Primary Neuronal Culture and Neuron/COS7 cell Co-culture experiments

Neuron/COS7 cell co-culture experiments were performed as described [20]. Primary hippocampal neuronal cultures from embryonic day 18 rats were used for synapse formation assays. Transfected COS7 cells were added to the transfected neurons at 9-11 days *in vitro*. After 1 day of co-culture, cells were fixed in cold 100% methanol and incubated with primary antibodies (anti-GFP to detect COS7 cells, anti-HA to detect ApoEr2 transfected COS7 cells, and synaptophysin to determine the recruitment of pre-synaptic marker) and secondary antibodies in PBS with 1% goat serum. 9–12 Random images were collected using a LSM510 confocal microscope (Carl Zeiss, Thornwood, NY, USA). Confocal z-series image stacks encompassing entire neurons were analyzed using MetaMorph software (Universal Imaging Corporation, Downingtown, PA, USA). Synapse formation was determined by quantifying co-immunostaining between the presynaptic marker synaptophysin and contacting axons of transfected COS7 cells. Presynaptic specializations (synaptophysin immunoreactivity) were normalized to the number of COS7 cells in the entire field (detected by immunostaining for GFP). Results were averaged for each condition (10 neurons per condition).

### Synaptosome fractionation

Synaptosome fractionation was performed as described [21], with minor modifications. Adult rat brains were briefly homogenized in 0.32 M sucrose, 4 mM HEPES-NaOH, pH 7.3 with protease inhibitors, and centrifuged at 1000×*g* for 10 min to recover the supernatant S1 and the pellet P1. S1 fraction was centrifuged at 12,000×*g* for 15 min to obtain the pellet P2 (crude synaptosome) and the supernatant S2. The P2 fraction was osmotically shocked by diluting with double-distilled water and further centrifuged at 25,000×*g* for 20 min to generate the pellet LP1 and the supernatant LS1. The LS1 fraction was subjected to ultracentrifugation to obtain the synaptic vesicle fraction LP2. LP1 was detergent extracted in buffer B (0.16 M sucrose, 5 mM Tris-HCl, pH 8.0, 0.5% Triton X-100, 0.5 mM β-ME, 1 mM EDTA, and protease inhibitors), and then centrifuged at 33,000×*g* for 20 min. The pellet LP1P was resuspended and applied to a discontinuous sucrose gradient consisting of 1.0 M, 1.5 M, and 2.0 M sucrose layers. After ultracentrifugation (200,000×*g* for 2 hr), the PSD fraction was recovered at the interface between 1.5 and 2.0 M sucrose.

### Golgi staining and analysis of dendritic morphology *in vivo*

Golgi staining was performed on ApoEr2 knockout mice and wild-type C57BL/6J mice (4 weeks or 1 year old; n = 4 per genotype and per age). For these experiments, we used the FD Rapid Golgi Stain kit (FD NeuroTechnologies, Ellicott City, MD) as described [22]. Freshly dissected brains were briefly immersed in solutions A and B for 2 weeks at room temperature and then transferred into solution C for 24 h at 4°C. The brains were sliced using a Vibratome (VT1000S; Leica, Germany) at a thickness of 150 µm. Bright-field microscopy (Axioplan 2; Zeiss, Brighton, MI) images (at 63× magnification) were taken of pyramidal neurons in cortical layers II/III (56 neurons per genotype per age). Images were coded, and dendritic spines counted in a blinded fashion using Neurolucida software (MicroBrightField, Williston, VA). All spines counted were also measured for spine length as described [23].

### Sholl analysis

To determine the effect of ApoEr2 on dendritic complexity, we co-transfected GFP-β-actin and ApoEr2-HA or GFP-β-actin and empty vector in primary hippocampal neurons (DIV 7). One week later, neurons were fixed in 4% paraformaldehyde and immunostained for GFP followed by DAB staining. Using Neurolucida software (MicroBrightField, Williston, VA), neurons were traced with the center of the soma as a focal point. Using Neuroexplorer software (Littleton, MA), we generated concentric circles around the center, beginning with a radius of 20 um, and increasing in size by 20 um increments. We then used this software to count the number of intersections on the perimeter of each circle per neuron, as well as to measure total dendritic length and dendritic number. A total of 10 neurons were used per condition in this analysis.

### Statistical analyses

All data were analyzed using either a 2-Tailed T-test or ANOVA with Graphpad Prism 4 software, using Tukey's Multiple Comparison test for post-hoc analyses with significance determined as p<0.05. Cumulative distribution plots were analyzed using the Kolmogorov-Smirnov test. Descriptive statistics were calculated with StatView 4.1 and expressed as mean ± S.E.M.

## Results

### ApoEr2 expression in synapses

**Figure 1:**
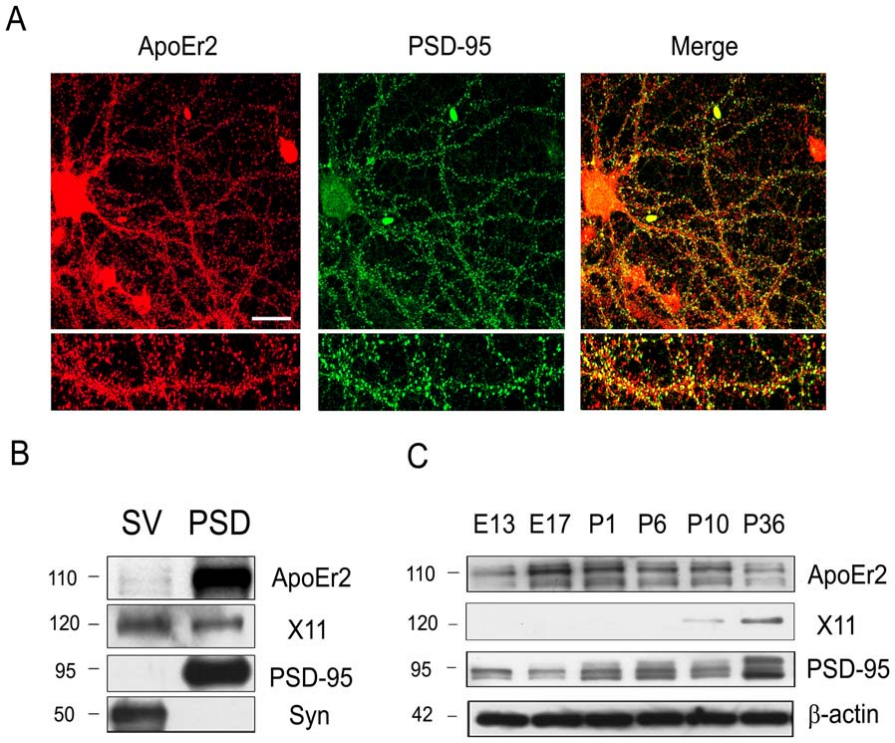
ApoEr2 is expressed at synapses. A. Primary hippocampal neurons at DIV 14 were immunostained for ApoEr2 and PSD-95. Primary antibodies were detected with Alexa Fluor 488 anti-rabbit (in green; left panel) and Alexa Fluor 594 anti-mouse (in red; middle panel). Immnolabeled neurons were imaged by confocal microscopy (63X). Colocalization of ApoEr2 and PSD-95 appears as yellow in the right panel. White bar represents 10 micrometers. B. We performed immunoblot analysis on ApoEr2, X11α, PSD-95, and synaptophysin in presynaptic vesicles (SV) and postsynaptic density (PSD) fractions. X11α is present in both pre- and postsynaptic fractions; synaptophysin is present in presynaptic fractions and PSD-95 is present in post-synaptic fractions. ApoEr2 is predominantly found in the postsynaptic fraction. C. Levels of ApoEr2, X11α, PSD-95, and β-actin (used as protein loading control), were examined by immunoblot at developmental stages (n = 3 per timepoint). ApoEr2 levels increased at E17-P6, a critical period for synaptogenesis.

ApoEr2 is expressed in neurons throughout the mammalian brain [24]. To determine whether ApoEr2 is expressed at the synapse, primary hippocampal neurons were immunostained with anti-ApoEr2 and anti-PSD-95 antibodies (Fig. 1A). ApoEr2 immunoreactivity was highest in pyramidal neurons, where it was found to have a punctate distribution throughout dendritic processes, and where it co-localized with the postsynaptic marker PSD-95, suggesting that ApoEr2 is present in synapses (Fig. 1A).

We also conducted synaptosomal fractionation on adult mouse forebrains. To monitor the purity of synaptosomal fractionations, we used PSD-95 as a postsynaptic marker and synaptophysin as a presynaptic marker. We then examined whether X11α and PSD-95, which interact with ApoEr2 [11], [12], are present in the pre- or postsynaptic fractions. We found that X11α was present in both pre- and postsynaptic fractions, while ApoEr2 and PSD-95 specifically localized to postsynaptic density fractions (Fig. 1B).

We next examined the expression levels of ApoEr2 and its cytoplasmic adaptor proteins during CNS development in mice. The levels of ApoEr2 in the brain increased markedly between embryonic day 17 and postnatal day 10 (Fig. 1C). X11α was detectable beginning at postnatal day 10 and increased until postnatal day 36; this period (postnatal day 10-36) is known to be important for synapse maturation (Fig. 1C). Levels of PSD-95 further increased between postnatal days 1 and 36, an important period for synaptogenesis, compared to embryonic stages (Fig. 1C). These data demonstrate age-dependent increases in levels of ApoEr2, PSD-95, and X11α, and reveal overlapping, but nonidentical, expression profiles among these proteins. The early peak in ApoEr2 levels suggests that it plays important roles in neuronal development, while sustained expression of ApoEr2 suggests its potentially ongoing involvement in adult plasticity.

### ApoEr2 promotes synapse formation in a heterologous co-culture system and dendritic spines in primary hippocampal neurons

**Figure 2:**
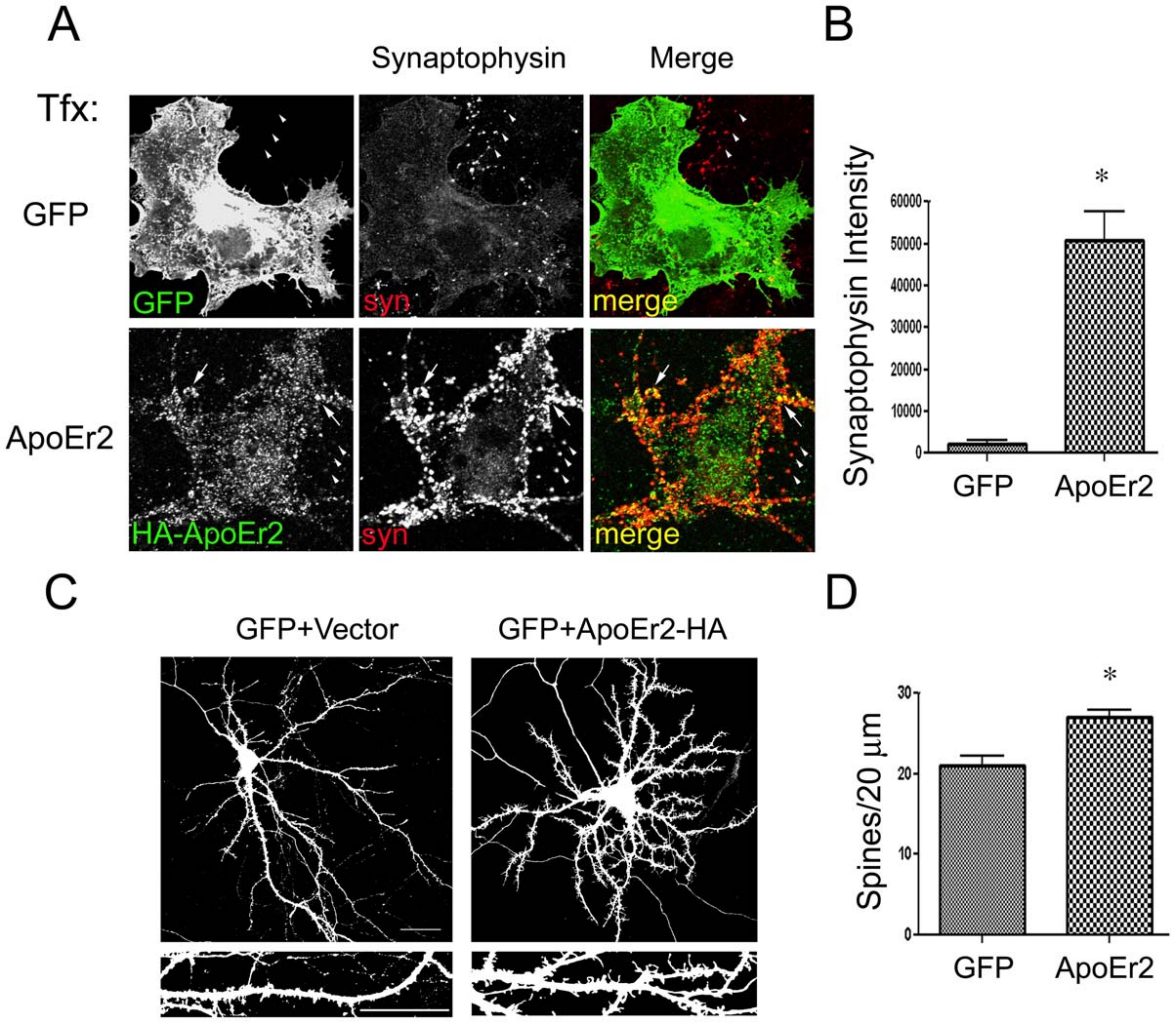
ApoEr2 promotes synapse formation in heterologous co-cultures and dendritic spine formation in primary hippocampal neurons. A. COS7 cells were transfected with GFP (upper panels) or ApoEr2-HA (lower panels) and then cultured with primary hippocampal neurons. ApoEr2 is expressed in clusters (lower panel left), in contrast to diffuse GFP distribution (upper left panel). Synaptophysin puncta in neuronal processes (middle panels) identified presynaptic sites (arrow heads, right panel). COS7 cells expressing ApoEr2 induced clustering of synaptophysin (arrows) compared to GFP alone. B. Quantification of average synaptophysin cluster intensity from the data in A (n = 10, *p<0.05). C. Cultured hippocampal neurons were transfected with GFP and empty vector or GFP and ApoEr2-HA as indicated. Morphology of neurons and dendritic spines were visualized by GFP fluorescence. Magnified examples of representative dendritic segments are shown in bottom panels. D. Quantification of spine density from C, with asterisks defining statistically significant differences from GFP-transfected cells (n = 15, *p<0.05). Error bars are represented as S.E.M. White bar represents 10 micrometers.

Culturing primary hippocampal neurons with heterologous cells transfected with candidate synaptogenic proteins has been used to analyze minimum requirements for synapse formation [20]. We examined whether synapse formation could be induced along hippocampal axons in a heterologous co-culture system by overexpressing ApoEr2 to mimic an artificial postsynaptic site. We measured average intensity of synaptophysin contacting transfected COS7 cells as an index of synaptogenesis and found that ApoEr2 alone induced dramatic accumulation of presynaptic specializations compared to GFP (Fig. 2A and B, lower panel), suggesting that ApoEr2 may be involved in synapse formation.

We then examined the postsynaptic effects of ApoEr2, specifically, the effects of ApoEr2 on dendritic spine formation. To test this, primary hippocampal neurons (transfected at DIV 14, expressed for 3 days) were transfected with GFP and empty vector or GFP and ApoEr2, and spine density was measured. We found that overexpression of ApoEr2 significantly increased spine number (20±3% increase) compared to GFP alone (n = 15) (Fig. 2C, D), suggesting that ApoEr2 plays a role in dendritic spine formation.

To further examine whether ApoEr2 regulates dendritic complexity, primary hippocampal neurons (DIV 7) were transfected with GFP-β-actin and empty vector or GFP-β-actin and ApoEr2-HA. We immunostained with GFP and then conducted DAB staining to examine neuronal morphology. Using sholl analysis, we measured dendritic complexity at incremental lengths from the soma. We found that overexpression of ApoEr2 did not increase dendritic complexity compared to controls (Fig. S1). Additionally, overexpression of ApoEr2 did not significantly affect total dendritic length or number compared to controls (data not shown). These data suggest that ApoEr2 does not alter dendritic complexity; however, it may regulate dendritic spine formation.

### The extracellular and intracellular domains of ApoEr2 are important for dendritic spine formation

**Figure 3:**
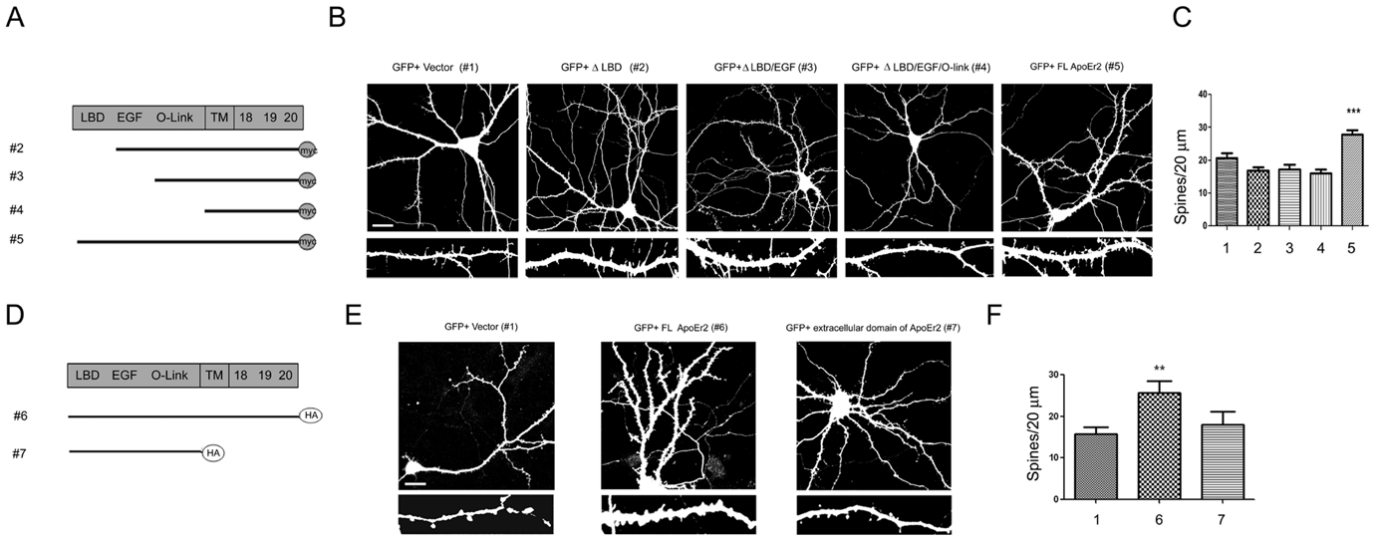
Extracellular and intracellular domains of ApoEr2 are essential for dendritic spine formation. A. Deletion constructs of ApoEr2 with a c-terminal myc tag. B. Primary hippocampal neurons (DIV 14) were transfected with GFP and empty vector (#1), GFP and ApoEr2 lacking a ligand binding domain (#2), GFP and ApoEr2 lacking ligand binding and EGF binding domains (#3), GFP and ApoEr2 lacking ligand binding, EGF binding and O-linked domains (#4) and GFP and full-length ApoEr2 (#5). After 48 hours, neurons were fixed and stained for GFP. Morphology of neurons and dendritic spines was visualized by GFP fluorescence. Magnified examples of representative dendritic segments are shown in bottom panels. C. Quantification of data shown in B (n = 15/per groups, ***, *p*<0.001). Error bars are represented as S.E.M. White bar represents 10 micrometers. D. Deletion constructs of ApoEr2 with a c-terminal HA tag. Primary hippocampal neurons (DIV 14) were transfected with GFP and empty vector (#1), GFP and full-length ApoEr2 (#6), or GFP and extracellular domain of ApoEr2 (#7). After 48 hours, neurons were fixed and stained for GFP. Morphology of neurons and dendritic spines was visualized by GFP fluorescence. Magnified examples of representative dendritic segments are shown in bottom panels. F. Quantification of data shown in C (n = 10/per group, **, *p*<0.005). Error bars are represented as S.E.M. White bar represents 10 micrometers.

Our previous findings demonstrated that ApoEr2 promotes dendritic spine formation (Fig. 2C, D). Therefore, we then examined which domains of ApoEr2 are essential to this process. To test this, we used deletion constructs of ApoEr2, as previously described (Fig 3A) [25], [26]. Primary hippocampal neurons were transfected with GFP and empty vector (#1), GFP and ApoEr2 lacking the ligand binding domain (#2), GFP and ApoEr2 lacking the ligand binding and EGF binding domains (#3), GFP and ApoEr2 lacking the ligand binding, EGF binding and O-linked domains (#4), and GFP and full-length ApoEr2 (#5), and spine density was measured (Fig 3B). Consistent with our previous findings, full-length ApoEr2 (#5) significantly increased dendritic spine density by 36% compared to GFP transfected cells (#1) (Fig. 2C, D, 3B, C). However, none of the constructs lacking the ligand binding domain of ApoEr2 increased dendritic spine formation (Fig. 3B, C). We further examined the specificity of the ligand binding domain of ApoEr2 in dendritic spine formation, we used a second set of deletion constructs of ApoEr2 with a C-terminal HA tag (Fig 3D). For these experiments, primary hippocampal neurons were transfected with GFP and empty vector (#1), GFP and a secreted form of ApoEr2 lacking the transmembrane and intracellular domains (#7), and GFP and full-length ApoEr2 (#6). Consistent with previous results, full-length ApoEr2 (#6) significantly increased dendritic spine density compared to GFP. Interestingly, the extracellular domain of ApoEr2 (#7) did not significantly increase dendritic spine density compare to controls, suggesting that the ligand binding domain of ApoEr2 alone is not sufficient to induce spine formation (Fig. 3E,F).

### ApoEr2 deficient mice have decreased spine density at 1 month of age

**Figure 4:**
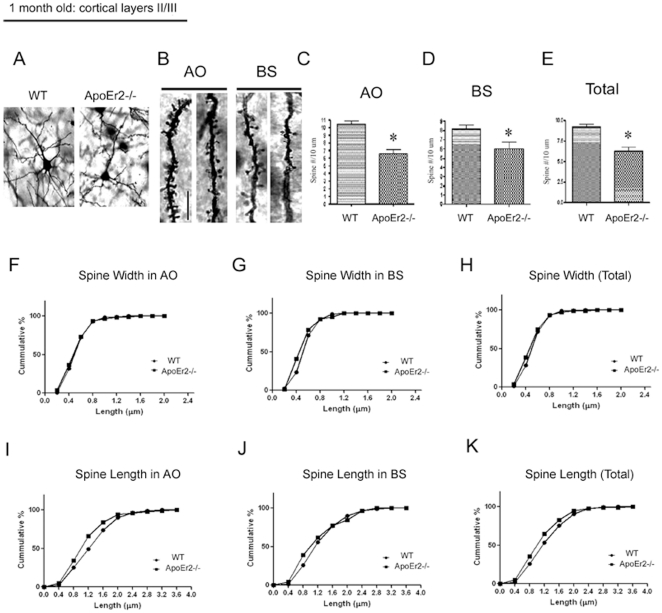
ApoEr2 deficient mice have reduced dendrite spine density in pyramidal neurons in cortical layers II/III at 1 month of age. Mouse brains were Golgi stained and cortical layers II/III imaged (*n* = 4 mice/genotype). A. Representative Golgi-impregnated pyramidal neurons in cortical layer II/III of a wild-type (left panel) and ApoEr2 knockout mice (right panel). B. Representative AO and BS dendrites per genotype. C–E. Averaged spine densities for each genotype. Black bar indicates 20 micrometer scale. C. Averaged spine density for AO spines (28 neurons/group). D. Averaged spine density for BS spines (28 neurons/group). E. Averaged total spine density, combining AO + BS dendrites (56 dendritic segments/group). Error bars are represented as S.E.M. *, *p*<0.05. F-H. The cumulative distribution percentage of spine width. There were no significant shifts in distribution between ApoEr2 knockout and wild-type littermates. I-K. The cumulative distribution percentage of spine length. ApoEr2 knockout mice had a significant shift to smaller spines compared to wild-type mice in the AO (I) and Total (K) but not BS (J). (*p*<0.05, Kolmogorov–Smirnov test).

To determine whether ApoEr2 has a similar effect on dendritic spine formation *in vivo*, we analyzed pyramidal neurons in cortical layers II/III using the rapid Golgi impregnation method in ApoEr2 knockout mice and wild-type littermates at 1 month old. Because spine density may vary within both the dendritic field of a single neuron and between different neurons [27], the spines of the pyramidal neurons were counted as apical oblique (AO), basal shaft (BS), and total (AO+BS) dendrites (Fig. 4A, B). We found that ApoEr2 knockout mice had significantly reduced dendritic spine number on AO, BS, and total dendrites compared with wild-type littermates at 1 month old (Fig. 4C–E).

Next, we examined the morphology of dendritic spines by measuring spine width and length in ApoEr2 knockout mice and wild-type littermates at 1 month old. The cumulative distributions revealed no significant differences in spine width between ApoEr2 knockout mice and wild-type mice (Fig 4F-H). However, we observed a significant shift in 1 month old ApoEr2 knockout mice to shorter spines compared to wild-type mice in the AO, but not BS dendrites (4I-K, p<0.05).

**Figure 5:**
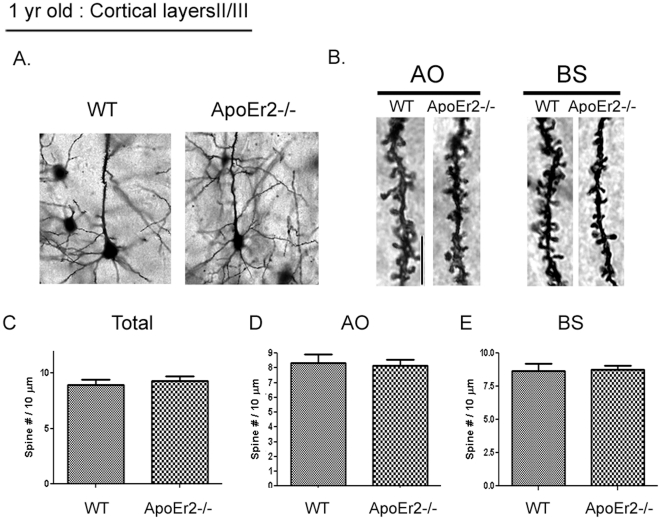
ApoEr2 deficient mice have no change in spine density in pyramidal neurons in cortical layers II/III at 1 year of age. Mouse brains were Golgi stained and neurons in cortical layers II/III imaged (*n* = 4 mice/genotype). A. Representative Golgi-impregnated pyramidal neuron in cortical layer II/III of a wild-type (left panel) and ApoEr2 knockout mice (right panel). B. Representative AO and BS dendrites per genotype. Black bar indicates 20 micrometer scale. C–E. Averaged spine densities for each genotype. C. Averaged total spine density, combining AO + BS dendrites (56 dendritic segments/group). D. Averaged spine density for AO spines (28 neurons/group). E. Averaged spine density for BS spines (28 neurons/group). Error bars are represented as S.E.M.

We further examined whether the effects of ApoEr2 on dendritic spine density was age-dependent. To test this, we conducted Golgi staining on ApoEr2 knockout mice and wild-type littermates at 1 year of age (Fig 5A, B). Interestingly, we observed no significant differences in dendritic spine densities between ApoEr2 knockout mice and wild-type littermates in cortical layers II/III at 1 year old (Fig 5C-E). This data suggests the existence of compensatory mechanisms between one month of age and one year of age in ApoEr2 knockout mice. We also conducted morphological analysis in 1 year old ApoEr2 knockout mice and wild-type littermates. The cumulative distributions revealed a significant shift in ApoEr2 knockout mice to shorter spines compared to wild-type mice in the AO, but not BS dendrites (data not show, p<0.05 for AO condition), consistent with our 1 month of age studies.

### ApoEr2 increased clustering of synaptic proteins

**Figure 6:**
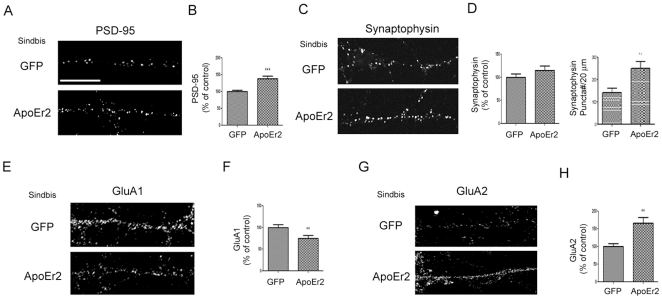
ApoEr2 regulates synaptic protein levels. A. Cultured hippocampal neurons (DIV 14) were infected with ApoEr2 or GFP sindbis virus for 20 hours and stained for PSD-95. B. Quantification of average puncta intensity in (A) (10 neurons/group, *** *p*<0.001). C. Cultured hippocampal neurons (DIV 14) were infected with ApoEr2 or GFP sindbis virus for 20 hours, and stained for synaptophysin. D. Quantification of average intensity (left panel) and puncta number (right panel) in (C) (10 neurons/group, ** *p*<0.005). E. Cultured hippocampal neurons (DIV 14) were infected with ApoEr2 or GFP sindbis virus for 20 hours and stained for GluA1. F. Quantification of average intensity in (E) (10 neurons/group, ** *p*<0.005). G. Cultured hippocampal neurons (DIV 14) were infected with ApoEr2 or GFP sindbis virus for 20 hours and stained for GluA2. H. Quantification of average intensity in (G) (10 neurons/group, ** *p*<0.005). Error bars are represented as S.E.M. White bar represents 10 micrometers.

Because our data indicate that ApoEr2 plays an important role in promoting synapse and dendritic spine numbers, we examined whether ApoEr2 affects clustering of synaptic proteins. For these experiments, primary hippocampal neurons were infected with ApoEr2 sindbis virus or GFP for 20 hours and intensity or puncta number of PSD-95 and synaptophysin were measured. We found that ApoEr2 significantly increased PSD-95 intensity throughout the cultures by 38% compared to GFP infected neurons (Fig 6A, B). We also found that, although ApoEr2 did not alter total synaptophysin levels, it did significantly increase puncta number of synaptophysin along the neuronal processes by 78% compared to control (Fig 6C, D). These data suggest that, while ApoEr2 does not immediately alter the level of synaptophysin, it does affect the localization of synaptophysin.

Because AMPA receptors are important for synaptic plasticity and dendritic spine formation, we examined whether ApoEr2 affected total levels of AMPA receptor subunits by infecting neurons with ApoEr2 or GFP [28], [29], [30]. We found that ApoEr2 infected neurons had significantly decreased total GluA1 intensity by 25% along the neuronal process (Fig 6E,F) and significantly increased total GluA2 intensity by 66% compared to GFP infected neurons (Fig 6G,H). These data suggest that ApoEr2 differentially regulates AMPA receptor subunit expression levels.

### ApoEr2 increases surface levels of GluA2 and decreases cell surface levels of GluA1

**Figure 7:**
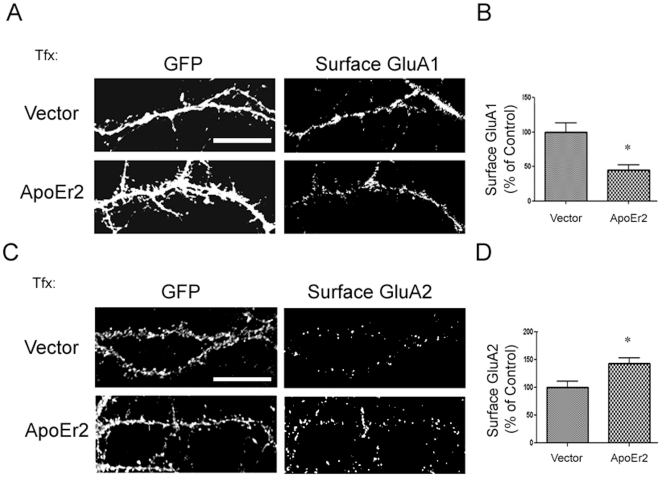
ApoEr2 decreases cell surface GluA1 and increases surface level of GluA2. A. Cultured hippocampal neurons (DIV 14) were transfected with GFP and empty vector or GFP and ApoEr2-myc for 48 hours and stained with an antibody recognizing the N terminus of GluA1 under impermeable conditions. B. Quantification of cell surface GluA1 levels in (A) (n = 15 neurons/group, * *p*<0.05). C. Cultured hippocampal neurons (DIV 14) were transfected with GFP and empty vector or GFP and ApoEr2-myc for 48 hours and stained with an antibody recognizing the N terminus of GluA2 under impermeable conditions D. Quantification of cell surface GluA2 levels in (C) (n = 15 neurons/group, * *p*<0.05). Error bars are represented as S.E.M. White bar represents 10 micrometers.

We then further examined whether ApoEr2 can regulate the distribution of AMPA receptor subunits on the cell surface. To test this, primary hippocampal neurons were transfected with GFP and empty vector or GFP and ApoEr2 and live cell surface staining was conducted. We found that ApoEr2 significantly decreased cell surface GluA1 by 65% (Fig 7A, B), but significantly increased cell surface of GluA2 by 43% (Fig 7C, D). These data further suggest that ApoEr2 differentially regulates AMPA receptor subunit distribution.

### X11α decreases cell surface ApoEr2 and inhibits ApoEr2 induction of synapse and spine formation

**Figure 8:**
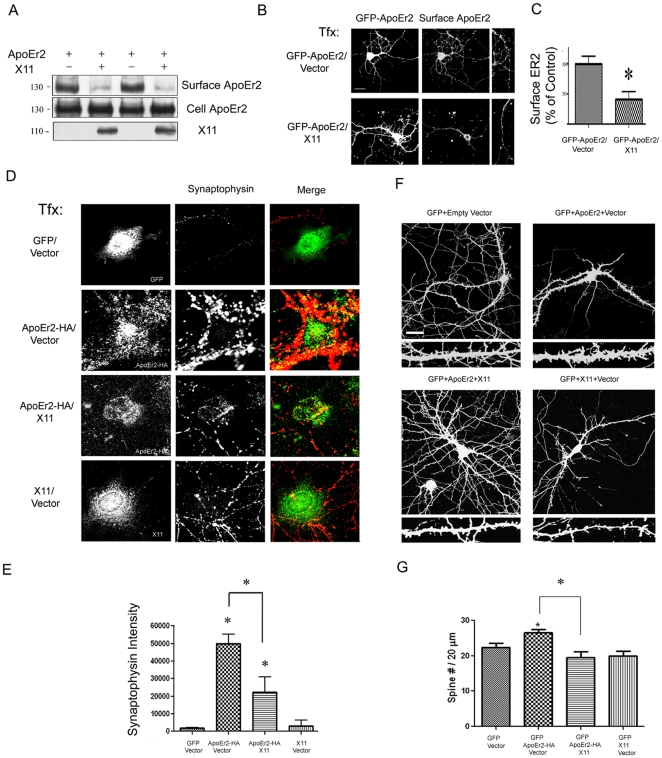
X11α decreases cell surface ApoEr2 and inhibits ApoEr2- induced synapse and dendritic spine formation. A. COS7 cells were transfected with GFP-ApoEr2 and vector (lane 1,3) or X11α (lane 2,4). Cell surface proteins were biotin-labeled, isolated with avidin-beads, and immunoblotted with GFP for ApoEr2. Full length X11α decreased surface levels of ApoEr2 (upper blot). Cell lysates showed similar levels of total ApoEr2 (lower blot). B. Cultured hippocampal neurons were transfected at DIV12 with either GFP-ApoEr2 and empty vector (upper panel) or GFP-ApoEr2 and X11α (lower panel). Surface ApoEr2 was measured with GFP at DIV14 by immunofluorescence of live cells. Left panels, GFP-ApoEr2; right panels, surface ApoEr2. C. Quantification of cell surface ApoEr2 intensity in neuronal processes in (B). The cell surface staining in neuronal processes showed a 54% decrease in ApoEr2 by X11α (n = 13, *p<0.05). D. COS7 cells were transfected with GFP and empty-vector (n = 10), ApoEr2-HA and empty vector (n = 15), ApoEr2-HA and X11α (n = 8), or X11α and empty vector (n = 14) and then cultured with primary hippocampal neurons. COS7 cells were stained for GFP in the GFP and empty vector condition, HA for the ApoEr2-HA and vector or ApoEr2-HA and X11 condition or X11 for the X11 and vector condition to identify transfected COS7 cells (left panel). Synaptophysin puncta in neuronal processes (middle panels) identified presynaptic sites. E. Quantification of average synaptophysin cluster intensity from data in D (*p<0.05). F. Cultured hippocampal neurons (DIV 14) were transfected with GFP and empty vector, GFP and empty vector and ApoEr2-HA, GFP and empty vector and X11α, or GFP and ApoEr2-HA and X11α as indicated. After 48 hours, morphology of neurons and dendritic spines were visualized by GFP fluorescence. Magnified examples of representative dendritic segments are shown in lower panels. G. Quantification of spine density from F, with asterisks defining statistically significant differences from GFP-transfected cells (n = 10, *p*<0.05). Error bars are represented as S.E.M. White bar represents 10 micrometers.

As described above, we and others demonstrated that ApoEr2 interacts with cytoplasmic adaptor proteins, specifically X11α and PSD-95 [7], [11], [12]. Initially, we wanted to determine whether the interaction between ApoEr2 and X11α affected cell surface levels of ApoEr2. To test this, COS7 cells were transfected with ApoEr2 and empty vector or ApoEr2 and X11α for 24 hours. Cell surface proteins were biotinylated, isolated with avidin beads, and immunoblotted for ApoEr2. We found that co-transfection with X11α decreased cell surface levels of ApoEr2 in COS7 cells (Fig 8A). Additionally, we conducted live cell surface staining by overexpressing ApoEr2 and empty vector or ApoEr2 and X11α in primary hippocampal neurons. We found that X11α decreased cell surface levels of ApoEr2 by 54% in primary hippocampal neurons (Fig. 8B, C, P<0.05; n = 13). Thus, two independent assays suggested that X11α can modulate the effects of ApoEr2 on cell surface expression.

Next, we examined whether the interaction between ApoEr2 and X11α could alter synapse formation. For these experiments, COS7 cells were transfected with GFP and empty vector, ApoEr2 and empty vector, ApoEr2 and X11α, or X11α and empty vector, and then cultured with primary hippocampal neurons as in Figure 2. Consistent with our previous findings, ApoEr2 significantly induced accumulation of presynaptic specializations compared to control vector (Fig. 2A, B, 8D, E). Interestingly, co-expression of X11α with ApoEr2 significantly inhibited accumulation of presynaptic specializations compared with ApoEr2 alone (Fig. 8D, E). However, X11α alone did not alter synaptophysin intensity (Fig. 8D, E). These data suggest that X11α can regulate the effect of ApoEr2 on synapse formation.

We also examined whether X11α could regulate the effect of ApoEr2 on dendritic spine formation in primary hippocampal neurons. To test this, primary hippocampal neurons (DIV 14) were transfected with GFP and empty vector, GFP with ApoEr2-HA and empty vector, GFP with ApoEr2-HA and X11α, or GFP with X11α and empty vector for 48 hours. After 48 hours, we conducted immunostaining with GFP (for morphological analysis). ApoEr2 significantly increased dendritic spine number (Fig. 8F and G, by 21%, n = 15, p<0.05) compared to GFP alone consistent with our earlier findings (Fig. 2). In addition, we found that co-transfection with ApoEr2 and X11α significantly reduced dendritic spine density (32% decrease, n = 15, p<0.05) compared to ApoEr2 alone (Fig. 8F and G). To ensure that expression of ApoEr2 was consistent across conditions, we repeated the experiment as described above. 48 hours later, we conducted immunostaining for anti-HA (to measure total levels of ApoEr2) and GFP (for morphological analysis). We observed no significant differences in total ApoEr2 levels across conditions (Fig. S2). These data suggest that ApoEr2 induces spine formation, but this function is regulated by interaction with X11α.

### PSD-95 increases cell surface ApoEr2 and enhances ApoEr2 induction of synapse and spine formation

**Figure 9:**
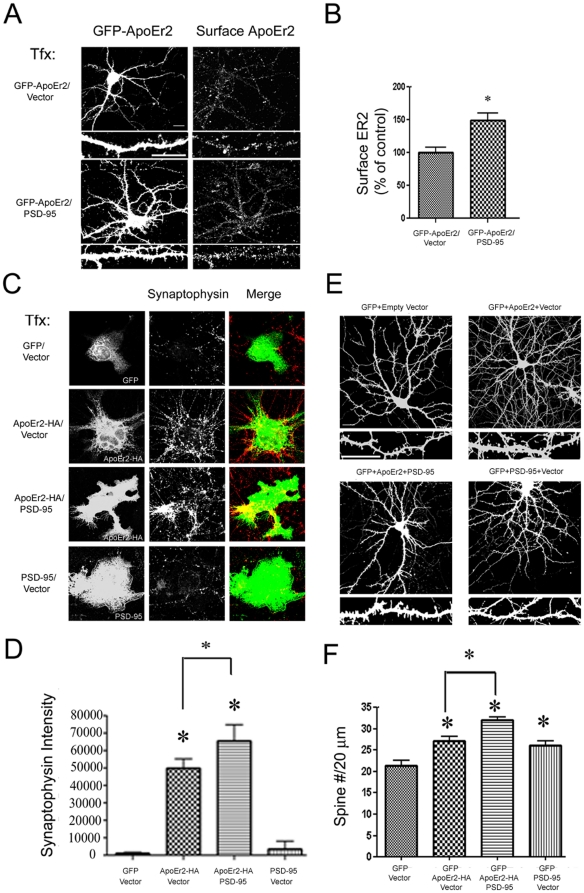
PSD-95 increases cell surface ApoEr2 and increases ApoEr2- induced synapse and dendrite spine formation. A. Cultured hippocampal neurons were transfected at DIV12 with either GFP-ApoEr2 and empty vector (upper panel) or GFP-ApoEr2 and PSD-95 (lower panel). Surface ApoEr2 was measured with GFP at DIV14 by immunofluorescence of live cells. Left panels, GFP-ApoEr2; right panels, surface ApoEr2. B. Quantification of surface ApoEr2 intensity in neuronal processes in (A). The cell surface staining in neuronal processes showed a 49% increase in ApoEr2 by PSD-95 (n = 10, *p<0.05). C. COS7 cells were transfected with GFP and empty vector, ApoEr2-HA and empty vector, ApoEr2-HA and PSD-95, or PSD-95 and empty vector and then cultured with primary hippocampal neurons. COS7 cells were stained for GFP in the GFP and empty vector condition, HA for the ApoEr2-HA and vector or ApoEr2-HA and PSD-95 condition or PSD-95 for the PSD-95 and vector condition to identify transfected COS7 cells (left panel). Synaptophysin puncta in neuronal processes (middle panels) identifies presynaptic sites. D. Quantification of average synaptophysin cluster intensity from data in (C) (n = 9/condition, *p<0.05). E. Cultured hippocampal neurons were transfected at DIV 14 with GFP and empty vector, GFP and ApoEr2 and empty vector, GFP and ApoEr2 and PSD-95, or GFP and empty vector and PSD-95 as indicated. 48 hours later, morphology of neurons and dendritic spines were visualized by GFP fluorescence. Magnified examples of representative dendritic segments are shown in lower panels. F. Quantification of spine density from (E), with asterisks defining statistically significant differences from GFP-transfected cells (n = 12, *p*<0.05). Error bars are represented as S.E.M. White bar represents 10 micrometers.

In our previous study, PSD-95 increased cell surface ApoEr2 levels in COS7 cells and in HEK293 cells [7]. For the present study, we further examined whether PSD-95 could alter cell surface levels of ApoEr2 in primary hippocampal neurons. To test this, primary hippocampal neurons were transfected with GFP-ApoEr2 fusion protein and vector or GFP-ApoEr2 and PSD-95 and live cell surface staining was conducted (Fig 9A). We found that PSD-95 significantly increased cell surface levels of ApoEr2 by 49% in primary hippocampal neurons compared to controls (Fig 9B).

We further examined whether PSD-95 can modulate ApoEr2-induced effects on synapse formation. COS7 cells were transfected with GFP and empty vector, ApoEr2 and empty vector, ApoEr2 and PSD-95, or PSD-95 and empty vector and then cultured with primary hippocampal neurons (Fig 9C). Co-expression of ApoEr2 and PSD-95 enhanced accumulation of presynaptic specializations compared to ApoEr2 alone (Fig. 9C, D).

Furthermore, we tested whether PSD-95 could regulate the effect of ApoEr2 on dendritic spine formation. To test this, primary hippocampal neurons (DIV 14) were transfected with GFP and empty vector, GFP and ApoEr2-HA and empty vector, GFP and ApoEr2-HA and PSD-95, or GFP and PSD-95 and empty vector. After 48 hours, we conducted immunostaining with anti-HA (to measure total levels of ApoEr2) and GFP (for morphological analysis). We found that ApoEr2 significantly increased dendritic spine density by 27% compared to GFP (Fig. 9E, F). Additionally, PSD-95 increased dendritic spine density by 21% compared to GFP – consistent with previous reports [31]. Interestingly, co-transfection of PSD-95 with ApoEr2 further increased the number of dendritic spines compared to ApoEr2 alone (Fig. 9E, F). ApoEr2 levels were consistent across all conditions (Fig. S2). These results suggest that interactions between ApoEr2 and PSD-95 can regulate synapse and spine formation by modulating surface ApoEr2 levels.

## Discussion

In the present study, we defined a physiological function of ApoEr2 at synapses. We demonstrated that ApoEr2 is expressed postsynaptically, and that ApoEr2 recruits and colocalizes with presynaptic specializations on contacting neuronal processes in COS7 cells. Moreover, ApoEr2 significantly increases dendritic spine density in primary hippocampal neurons, suggesting that ApoEr2 also contributes to spine development. We also examined how interaction between ApoEr2 and the synaptic adaptor proteins X11α and PSD-95 modulated ApoEr2-induced effects on synapse and dendritic spine formation. We found that X11α decreased, while PSD-95 increased, cell surface ApoEr2 levels. Additionally, we found that X11α inhibited, while PSD-95 enhanced ApoEr2-induced effects on synapses and spines. These results demonstrate that ApoEr2 is important for dendritic spine formation, and that this effect can be further modulated via interaction with its cytoplasmic adaptor proteins.

ApoEr2 plays an important role in induction of LTP, learning and memory, and synaptic transmission in adult brain [5], [32]. These processes require the cytoplasmic, alternatively spliced exon 19 of ApoEr2 [5]. In this study, we demonstrated the ability of ApoEr2 to recruit and colocalize with presynaptic specializations on contacting neuronal dendrites (Fig. 2), suggesting that ApoEr2 may be involved in synapse formation. This recruitment is often due to a trans-synaptic interaction involving either a homophilic or heterophilic adhesion [33]. Since we observed that ApoEr2 induced synapse formation, we hypothesize that presynaptic Amyloid Precursor Protein (APP), which is expressed pre-and postsynaptically and known to associate with ApoEr2 [26], [34], [35], or other presynaptic proteins may form trans-synaptic interactions with postsynaptic ApoEr2 to regulate synapse formation. There may also be extracellular molecules, such as F-spondin [26], [36] or Reelin [22], that affect the trans-synaptic interaction between ApoEr2 and pre-synaptic proteins to further regulate synapse formation. Additionally, it is possible that intracellular molecules, X11α and PSD-95, may alter ApoEr2's effects on synapse formation, as the present study demonstrates. However, further systematic analysis is necessary to determine the exact mechanism by which ApoEr2 acts.

We observed that ApoEr2 promotes dendritic spine formation in primary hippocampal neurons. To examine the effects of ApoEr2 on dendritic spine formation *in vivo*, we conducted a Golgi rapid impregnation staining in ApoEr2 knockout mice and wild-type littermates at 1 month and 1 year of age. We found that 1 month old ApoEr2 knockout mice had significant deficits in spine number compared to controls (Fig. 4). However, 1 year old ApoEr2 knockout mice had similar spine densities compared to controls, suggesting that there may be compensatory mechanisms following initial spine formation in ApoEr2 knockout mice. One possible compensatory mechanism is modulation of extracellular ligands for ApoEr2, such as apolipoprotein E (apoE) or Reelin, which are known to be important for dendritic spine formation [22], [37], [38], [39]. Perhaps apoE and Reelin levels increased to compensate for ApoEr2 deficiency in ApoEr2 knockout mice, resulting in the similar spine densities compared to wild-type littermates that we observed at 1 year. It is also possible that ApoEr2 plays an important role in the initial formation of dendritic spines, but not their maintenance in adulthood. These possibilities can be further examined in the study of this receptor and its ligands as a function of age.

In the present study, we examined how ApoEr2 affects synapse formation by measuring synaptic protein levels of synaptophysin and PSD-95 in ApoEr2-infected neurons compared to controls. We observed that ApoEr2 infected neurons increased levels of PSD-95 and increased puncta of synaptophysin. Additionally, ApoEr2 overexpression increased protein levels of SPAR, a Rap-specific GAP that promotes spine formation (data not shown) [23], [40]. These data suggest that ApoEr2 may alter the number of synaptic sites in cultured hippocampal neurons by regulating the assembly of a complex of postsynaptic proteins. We also examined the effects of ApoEr2 on total and cell surface levels of AMPA receptor subunits and observed that ApoEr2 caused an increase in total and cell-surface levels of GluA2, but a decrease in total and cell surface levels of GluA1. These results are somewhat surprising given that GluA1/A2 heteromers comprise the predominant AMPA receptor population in the hippocampus [41], [42]. Our findings suggest that ApoEr2 may alter the subunit composition of AMPA receptors from a GluA1/A2 to a GluA2/A3 combination. While neither GluA1 nor GluA2 is absolutely required for normal dendritic spine formation or morphogenesis *in vivo* [43], [44], GluA2 overexpression has been shown to enhance dendritic spine formation in hippocampal cultured neurons [30]. Thus, the preferential increase in GluA2 by ApoEr2 could reflect a mechanism to stimulate spinogenesis during development.

Alternatively, a shift from GluA1 to GluA2-containing receptors could be related to differential cell biological properties of the two receptor subunits. For example, GluA1 has been suggested to require activity-dependent stimulation for acute delivery, whereas GluA2 may undergo constitutive trafficking and turnover [28], [45]. In addition, GluA1 channel conductance or opening is enhanced by phosphorylation during various forms of synaptic plasticity such as LTP [46]. Furthermore, the great majority of GluA2 exists in a calcium impermeable isoform, such that regulation of GluA2 levels can serve as a mechanism to limit calcium permeability of AMPA receptors [47]. Taken together, these divergent properties suggest that decreased GluA1 may lead to a reduced ability to undergo activity-dependent synaptic potentiation, perhaps as a compensatory response to the increase in number of excitatory synapses and dendritic spines generated by elevated GluA2. A better understanding of the functional consequences of AMPA receptor subunit composition shifts observed here will require additional studies conducting detailed electrophysiological analysis of ApoEr2-expressing neurons.

The definitive mechanism by which ApoEr2 acts remains unknown. It is possible that ApoEr2 modulates dendritic spine morphology by promotion of de novo spine formation or by stabilization of existing dendritic spines. We hypothesize that ApoEr2 may regulate spine formation through interaction with Reelin [38] as well as through interaction with cytoplasmic adaptor proteins X11α and PSD-95. Supporting this hypothesis, we observed that the ligand binding domain of ApoEr2 is essential, but not sufficient for spine formation, suggesting that extracellular interaction with Reelin may be part of the mechanism by which ApoEr2 increases dendritic spine number. Furthermore, we found that co-transfection with ApoEr2 and PSD-95 enhanced dendritic spine formation as compared to ApoEr2 alone, as opposed to co-transfection with ApoEr2 and X11α, which decreased spine formation as compared to ApoEr2 alone. However, further investigation is necessary to determine how ApoEr2 affects synapses and spine formation.

In conclusion, our results demonstrate, for the first time, that ApoEr2 promotes presynaptic differentiation and dendritic spine formation *in vitro* and *in vivo*, an effect further regulated by interaction with cytoplasmic adaptor proteins. These findings provide a better understanding of the physiological actions of ApoEr2 in the normal brain. We speculate that these molecular roles may be relevant to ApoEr2 functions in synaptic plasticity and learning and memory, both of which depend on the dynamic or persistent formation or elimination of synapses and dendritic spines.

## Supporting Information

Figure S1 **ApoEr2 does not alter dendritic complexity compared to controls**. Primary hippocampal neurons (DIV 7) were transfected with GFP-β-actin and empty vector or GFP-β-actin and ApoEr2-HA. On DIV 14, neurons were immunostained with GFP followed by DAB to visualize cell morphology. A. Three-dimensional graphical tracing representing dendrite morphology for control (left panel) and ApoEr2 (right panel) conditions. Line bar represents 50 µm length. C. Using sholl analysis of cells in A, we graphed the average intersection per shell per neuron against the distance from the soma (in micrometers).
(TIF)

Figure S2 **ApoEr2 total expression levels were not significantly different between neurons across conditions.** Primary hippocampal neurons (DIV 14) were transfected with GFP and empty vector, GFP with ApoEr2-HA and empty vector, GFP with ApoEr2-HA and X11α, GFP with X11α and empty vector, GFP with PD95 and empty vector or GFP with PSD95 for 48 hours. HA staining was performed to measure total levels of ApoEr2 and GFP staining was performed for morphological analysis. A. Representative images for the conditions indicated. Primary antibodies were detected with Alexa Fluor 488 anti-rabbit for GFP (top panel) and Alexa Fluor 594 anti-mouse for HA (bottom panel). Immunolabeled neurons were imaged by confocal microscopy (63X). White bar represents 20 micrometers in length. B. Quantification of average HA intensity from (A). Error bars are represented as S.E.M.
(TIF)
