# Supplementary figures and images for: ApoE Receptor 2 Regulates Synapse and Dendritic Spine Formation
Source: PLoS One. 2011 Feb 15;6(2):e17203. doi: 10.1371/journal.pone.0017203 (PMC3039666; doi:10.1371/journal.pone.0017203)

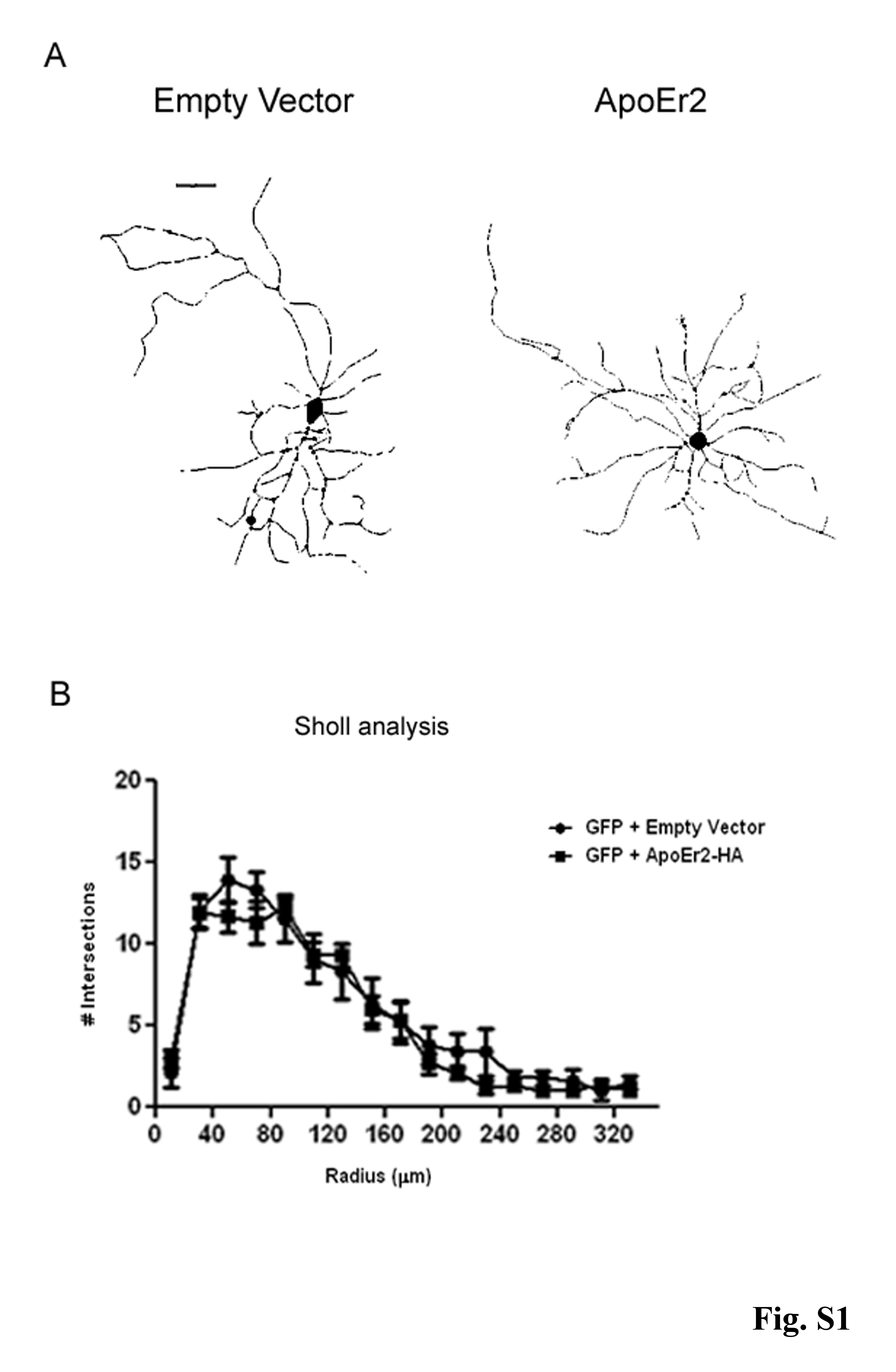

Supplement: Figure S1 — ApoEr2 does not alter dendritic complexity compared to controls. Primary hippocampal neurons (DIV 7) were transfected with GFP-β-actin and empty vector or GFP-β-actin and ApoEr2-HA. On DIV 14, neurons were immunostained with GFP followed by DAB to visualize cell morphology. A. Three-dimensional graphical tracing representing dendrite morphology for control (left panel) and ApoEr2 (right panel) conditions. Line bar represents 50 µm length. C. Using sholl analysis of cells in A, we graphed the average intersection per shell per neuron against the distance from the soma (in micrometers). (TIF) [file pone.0017203.s001.tif]

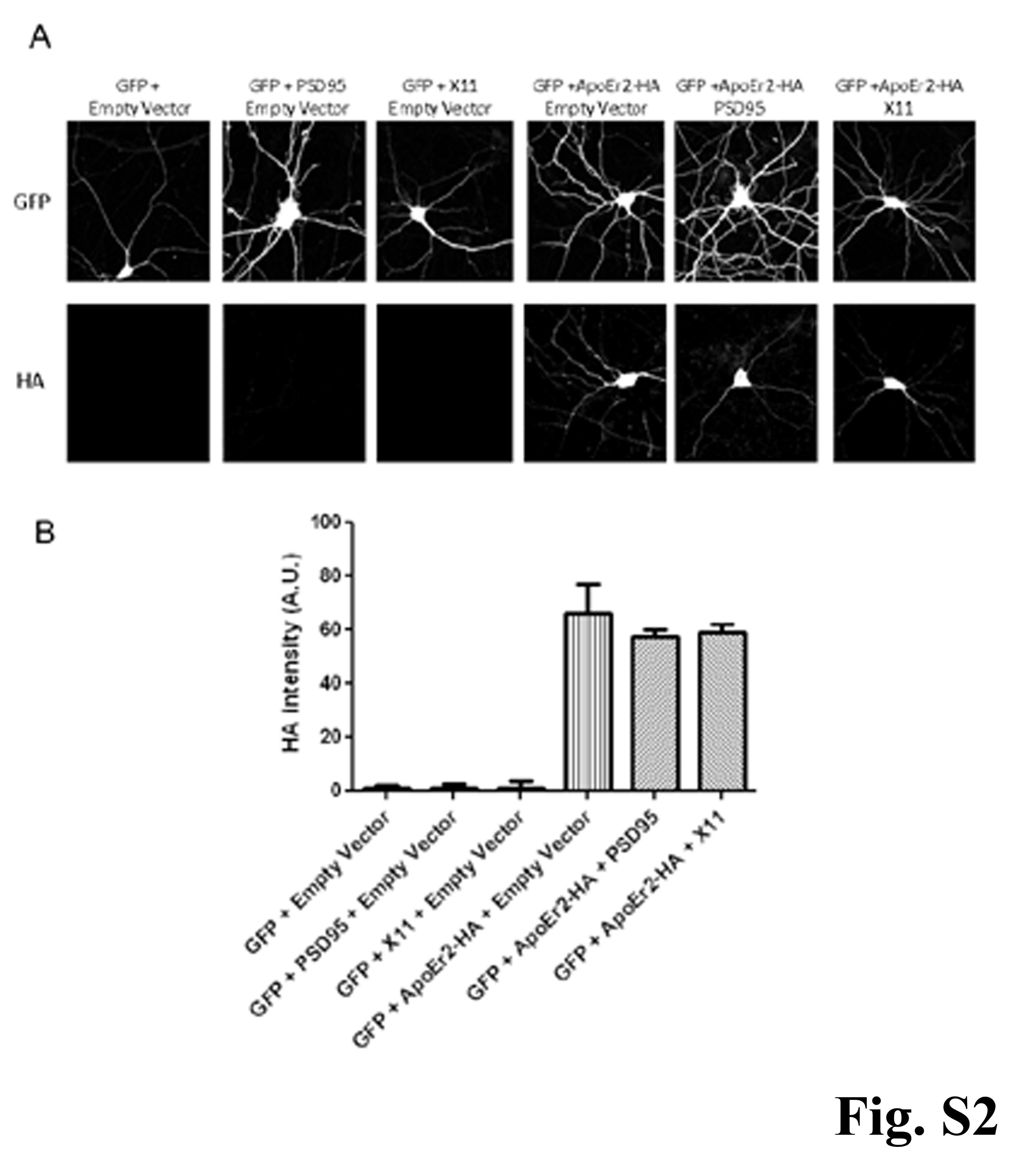

Supplement: Figure S2 — ApoEr2 total expression levels were not significantly different between neurons across conditions. Primary hippocampal neurons (DIV 14) were transfected with GFP and empty vector, GFP with ApoEr2-HA and empty vector, GFP with ApoEr2-HA and X11α, GFP with X11α and empty vector, GFP with PD95 and empty vector or GFP with PSD95 for 48 hours. HA staining was performed to measure total levels of ApoEr2 and GFP staining was performed for morphological analysis. A. Representative images for the conditions indicated. Primary antibodies were detected with Alexa Fluor 488 anti-rabbit for GFP (top panel) and Alexa Fluor 594 anti-mouse for HA (bottom panel). Immunolabeled neurons were imaged by confocal microscopy (63X). White bar represents 20 micrometers in length. B. Quantification of average HA intensity from (A). Error bars are represented as S.E.M. (TIF) [file pone.0017203.s002.tif]
